# A new species of Ceratina (Ceratinula) Moure, 1941, with notes on the taxonomy and distribution of Ceratina (Ceratinula) manni Cockerell, 1912, and an identification key for species of this subgenus known from Brazil (Hymenoptera, Apidae, Ceratinini)

**DOI:** 10.3897/zookeys.1006.57599

**Published:** 2020-12-23

**Authors:** Favízia Freitas de Oliveira, Lívia Raquel de Sousa Silva, Fernando César Vieira Zanella, Caroline Tito Garcia, Heber Luiz Pereira, Claudia Quaglierini, Camila Magalhães Pigozzo

**Affiliations:** 1 Laboratório de Bionomia, Biogeografia e Sistemática de Insetos (BIOSIS), Instituto de Biologia, Universidade Federal da Bahia (IBIO-UFBA), Rua Barão de Jeremoabo, número 668, Campus Universitário de Ondina, CEP: 40170-115, Salvador, Bahia, Brazil Universidade Federal da Bahia Salvador Brazil; 2 Centro Universitário Jorge Amado (UNIJORGE), Av. Luis Viana, n. 6775, Paralela, CEP: 41.745-130, Salvador, Bahia, Brazil Centro Universitário Jorge Amado Salvador Brazil; 3 Instituto Latino Americano de Ciências da Vida e da Natureza, Universidade Federal da Integração Latino-Americana, Avenida Silvio Américo Sasdelli, número 1842, Bairro Itaipu A, Edifício Comercial Lorivo, CEP: 85866-000, Caixa Postal 2044 – Foz do Iguaçu, Paraná, Brazil Universidade Federal da Integração Latino-Americana Foz do Iguaçu Brazil; 4 Programa de Pós-Graduação em Zootecnia, Departamento de Zootecnia, Centro de Ciências Agrárias, Universidade Estadual de Maringá, Avenida Colombo, 5790, 87020-900, Maringá, Paraná, Brazil Universidade Estadual de Maringá Maringá Brazil; 5 Tropical Intelligence Manager, Bayer SA – Brasil, Rua Domingos Jorge, 1100 | 504 |3 andar, São Paulo/SP, Brazil Bayer SA – Brasil São Paulo Brazil

**Keywords:** Anthophila, biogeography, small carpenter bees, South American diagonal of open formations, taxonomy

## Abstract

A new species of the small carpenter bee, genus Ceratina (Ceratinula) Moure, from the Cerrado Biome in midwestern Brazil is described and illustrated. Ceratina (Ceratinula) fioreseana Oliveira, **sp. nov.** is easily distinguished from its congeners by the size of the facial maculations and the honey-yellow color of the legs and antennal scape, which distinguish it especially from Ceratina (Ceratinula) manni Cockerell, 1912, the most similar species in terms of facial maculation patterns. The geographic records of *C. manni*, here interpreted as endemic to the semiarid Caatinga region in northeastern Brazil, are presented, with new records for the Brazilian states of Piauí, Ceará and Bahia. A morphological description of both species is provided, including a comparison with the type specimen of *C. manni* from the state of Paraíba (Guarabira, formerly named Independencia). An identification key is provided for the described species of Ceratina (Ceratinula) recorded for Brazil according to Moure’s Catalogue of Neotropical Bees.

## Introduction

Bees (Hymenoptera, Anthophila) are a diverse group of insects, with more than 20,000 described species and many more estimated to exist (Michener 2007; Discover Life 2020; ITIS 2020). Bees have established close relationships with angiosperms during the evolution of the two groups, and the majority of species feed exclusively on floral resources, visiting and pollinating flowers (Pinheiro et al. 2014). Their lifestyles range from solitary to social. The social species are the best known and studied, because many are used commercially, such as honeybee *Apis mellifera* Linnaeus, 1758 and “stingless bees” (Meliponini) in South America. Although less familiar to the general public, solitary bees represent the vast majority of bee species worldwide, comprising ca. 85% of the world apifauna (Batra 1984). Solitary bees act as key pollinators in natural and agricultural ecosystems (Roubik 1995; Garibaldi et al. 2013).

The small carpenter bees comprise a group of mainly solitary bees, with only a single genus, *Ceratina* Latreille 1802 (Ceratinini) (Rehan 2020). They are closely related to the well-known large carpenter bees or solitary “mamangavas” (genus *Xylocopa* Latreille, 1802, Xylocopini), in the subfamily Xylocopinae, together with Manuelini and Allodapini (Michener 2007; Flores-Prado et al. 2010). In the Catalog of Neotropical Bees, which uses the classification of Moure et al. (2012), these taxa are treated as subtribes of Xylocopini, namely: Ceratinina, Xylocopina, and Manuellina (the allodapines are excluded as they do not occur in the New World). The small carpenter bees nest in dead broken stems of pithy plants (Rehan 2020).

The genus *Ceratina* is cosmopolitan and highly diverse, although in Australia the group is rare and limited in distribution (Michener 2007). The genus has ca. 380 species recognized as valid (Discover Life 2020; ITIS 2020), distributed in 23 documented subgenera (Michener 2007; Terzo et al. 2007; Roig-Alsina 2013), six of these occurring in the New World (Table 1). Among the Neotropical subgenera of *Ceratina*, five have been recorded in Brazil, and the sixth was recently described as new to science (Roig-Alsina 2013 – new subgenus with 11 species, eight of which were new to science).

**Table 1.** The Subgenera of *Ceratina* Latreille, 1802*.

Subgenera		Species number	Geographic distribution
01	*Calloceratina* Cockerell, 1924	15	**New World**: Neotropical Region (Belize, Bolivia, Brazil, Colombia, Costa Rica, Ecuador, French Guiana, Guatemala, Guyana, Honduras, Mexico, Panama, Paraguay, Trinidad and Tobago and Venezuela) and marginally in Nearctic Region (Mexico).
02	*Catoceratina* Vecht, 1952	2	**Old World**: Burma, Thailand, and the Philippine Islands south to Sumatra, Java, and Borneo.
03	*Ceratinas. str.* Latreille, 1802	29	**Old World**: France to Turkey, and south through Africa to South Africa, and east through Eurasia to Japan, and Thailand.
04	*Ceratinidia* Cockerell & Porter, 1899	50	**Old World**: Sri Lanka and India throughout southeastern Asia, north through China to the maritime province of Siberia, also including all of Japan, Taiwan, the Philippines, and Indonesia east to the western tip of New Guinea.
05	*Ceratinula* Moure, 1941	37	**New World**: Nearctic Region: United States of America (Florida, Georgia, Louisiana, North Carolina, Texas); Neotropical Region: Argentina (Misiones); Bahamas; Belize; Bolivia; Brazil (Amazonas, Ceará, Maranhão, Minas Gerais, Paraná, Paraíba, Pará, Rio de Janeiro, São Paulo); Costa Rica (Alajuela, San José); Cuba; Dominican Republic; Haiti; Honduras; Panama (Chiriquí, Coclé, Panamá); Paraguay; Peru (Loreto); Saint Vincent and the Grenadines; Trinidad and Tobago.
06	*Chloroceratina* Cockerell, 1918	2	**Old World**: From northern Luzon, in the Philippine Islands.
07	*Copoceratina* Terzo & Pauly, 2001	2	**Old World**: From Kenya to South Africa, Madagascar and Seychelles.
08	*Crewella* Cockerell, 1903	32	**New World**: Neotropical Region: Argentina, Bolivia, Brazil, Colombia, French Guiana, Guyana, Panama, Paraguay, Uruguay, Venezuela.
09	*Ctenoceratina* Daly & Moure, 1988	10	**Old World**: Africa, from Senegal to Ethiopia, south to South Africa.
10	*Dalyatina* Terzo, 2007	7	**Old World**: From Spain, France, Croatia, Greece, Turkey, Turkmenistan to subsaharian Africa.
11	*Euceratina*Hirashima, Moure & Daly, 1971	38	**Old World**: From Britain, Spain, and Morocco east through Europe and the Mediterranean basin to southern Russia, Pakistan, and Somalia.
			One species, the parthenogenetic *Ceratina dallatorreana* Friese, is established in California, introduced by commerce.
12	*Hirashima* Terzo & Pauly, 2001	7	**Old World**: From Tanzania and Nigeria south to South Africa, and Madagascar and Aldabra.
13	*Lioceratina* Vecht, 1952	8	**Old World**: From India through Southeast Asia, Indonesia as far east as Bali, Sulawesi, and Philippines.
14	*Malgatina* Terzo & Pauly, 2001	1	**Old World**: Madagascar.
15	*Megaceratina*Hirashima, 1971	1	**Old World**: Africa, from Senegal to Zaire and east to Uganda.
16	*Neoceratina* Perkins, 1912	13	**Old World**: From Turkey and Cyprus east through southwest and southern Asia and Indonesia to southern China, the Ryukyu Islands, Micronesia, Philippines, and south to the Bismarck Archipelago, the Solomon Islands, and eastern Australia as far as New South Wales.
			One species was introduced in Hawaii (Snelling 2003).
17	*Neoclavicera* Roig-Alsina, 2013	11	**New World**: South America: from Argentina, Uruguay, Brazil, Paraguay, Bolivia and Peru.
18	*Pithitis* Klug, 1807	25	**Old World**: Africa: from Senegal to Egypt south throughout Africa to Cape Province; Crete, eastward in Saudi Arabia, Yemen, Pakistan, India, and SriLanka, throughout southeast Asia to Philippines, Taiwan, Ryukyu Islands, southeast China, and through Indonesia east as far as Ambon.
			One species was introduced in Hawaii (Snelling 2003).
19	*Protopithitis*Hirashima, 1969	2	**Old World**: Africa: Gabon, Congo, Angola, Zambia, Zaire, Tanzania, Mozambique, South Africa.
20	*Rhysoceratina* Roig-Alsina, 2013	10	**New World**: South America: Argentina, Uruguay, Brazil, Paraguay, Colombia and Venezuela.
21	*Simioceratina* Daly & Moure, 1988	3	**Old World**: Liberia to Kenya south to Namibia and Natal Province, South Africa.
22	*Xanthoceratina* Vecht, 1952	7	**Old World**: Sri Lanka, Burma and Southeast Asia, including Indonesia as far east as Java, Philippines, and southern China.
23	*Zadontomerus* Ashmead, 1899	29	**New World**: From Quebec and British Columbia in Canada to south throughout North and Central America until northern Colombia and Venezuela.
Total of placed species		338	Several other species have not been yet placed into any subgenus.

*According to Snelling (2003), Terzo et al. (2007), Michener (2007), Moure (2012), Roig-Alsina (2013), Roig-Alsina (2016), Flórez-Gómez and Griswold (2020), Discover Life (2020).

Rehan and Sheffield (2011) also described a new cryptic species from eastern North America based on integrative taxonomic studies, and Flórez-Gómez and Griswold (2020) described a new species endemic to the Caribbean Region of Colombia and Venezuela, showing that the global diversity in the genus is undoubtedly underestimated.

Some investigators have considered the genus *Ceratina* as a key taxon for understanding the transition from subsocial to social behavior, as they have a broad range of social behaviors, ranging from solitary, subsocial, and semisocial to eusocial colony organization (Rehan 2020). Rehan et al. (2015) provided evidence of nest reuse consistent with the hypothesis of kin associations for three Neotropical species of Ceratina (Ceratinula) from Panama, confirming that sociality occurs in low frequency in *Ceratina* bee populations, generally in a third or less of a population. According to Rehan et al. (2015), the solitary nature of the majority of colonies indicates that solitary nesting is adaptive in the species studied by these authors.

Of the five subgenera with species recorded in Brazil, Ceratina (Ceratinula) Moure, 1941 is the most diverse, with 37 species, followed by: C. (Crewella) Cockerell, 1903 (32 species); C. (Calloceratina) Cockerell, 1924 (15 species); C. (Neoclavicera) Roig-Alsina, 2013 (11 species); and C. (Rhysoceratina) Michener, 2000 (10 species) (Moure 2012; Roig-Alsina 2013; Roig-Alsina 2016; Discover Life 2020; Flórez-Gómez and Griswold 2020). Ceratina (Ceratinula) occur throughout the Neotropical region and extend to the United States of America (Silveira et al. 2002; Michener 2007; Moure 2012). Despite the fact that 14 of its 37 valid species occur in Brazil (Moure 2012; Table 2), no taxonomic review that includes the Brazilian species has yet been published. Moure (1941: 78–83), when describing *Ceratinula* as a new genus, redescribed or added taxonomic information for eight Neotropical species (six from the Brazilian fauna, not including *C. manni*) and described five new species, including four from Brazil.

**Table 2.** Species of Ceratina (Ceratinula) Moure, 1941 recorded in Brazil according to “Catalogue of Bees (Hymenoptera, Apoidea) in the Neotropical Region – online version” (Moure 2012).

Species		Type locality* and additional geographic records**	Domain
1	Ceratina (Ceratinula) augochloroides Ducke, 1910	*Serra de Baturité (Ceará, Brazil)	Rain Forest highland enclave in Caatingas
		#Manaus (Amazonas, Brazil)	
2	Ceratina (Ceratinula) biguttulata (Moure, 1941)	*Curitiba (Paraná, Brazil)	Atlantic Rain Forest
		#São Paulo (São Paulo, Brazil), Rio Grande do Sul (Brazil)	
3	Ceratina (Ceratinula) combinata Friese, 1910	*Belém (Pará, Brazil)	Amazonian
4	Ceratina (Ceratinula) fulvitarsis Friese, 1925	*São Paulo (São Paulo, Brazil)	Atlantic Rain Forest
5	Ceratina (Ceratinula) immaculata Friese, 1910	*Belém, Itaituba (Pará, Brazil)	Amazonian
6	Ceratina (Ceratinula) lucidula Smith, 1854	*Santarém (Pará, Brazil)	Amazonian and Atlantic Rain Forest
		*(synonymous): São Paulo (São Paulo, Brazil)	
		**Ceará, Minas Gerais, Pará, São Paulo (Brazil), Paraguay	
	It is possible that different species are being considered as *C. lucidula*.	#Rio de Janeiro, Paraná, Rio Grande do Sul (Brazil)	
7	Ceratina (Ceratinula) manni Cockerell, 1912	*Guarabira (Paraíba, Brazil)	Caatingas
		#São Paulo (Brazil)	
8	Ceratina (Ceratinula) melanochroa (Moure, 1941)	*Curitiba (Paraná, Brazil)	Atlantic Rain Forest
		#Rio Grande do Sul (Brazil)	
9	Ceratina (Ceratinula) minima Friese, 1908	*Trinidad	Caribe-Guajira Subequatorial and Amazonian
		**Trinidad and Tobago, Brazil	
		# Merida (Venezuela)	
10	Ceratina (Ceratinula) muelleri Friese, 1910	*Belém, Óbidos (Pará, Brazil)	Amazonian and Atlantic Rain Forest
	It is possible that different species are being considered as *C. muelleri*. The diagnosis presented in the key is based on specimens collected in one of the type locality (Belém, Pará, Brazil), which fit perfectly with the original description of the species.	**Misiones (Argentina), Amazonas, Ceará, Maranhão, Minas Gerais, Paraná, Pará, Rio de Janeiro, São Paulo (Brazil)	
		# Trinidad and Tobago, Rio Grande do Sul (Brazil)	
11	Ceratina (Ceratinula) piracicabana Schrottky, 1910	*Piracicaba (São Paulo, Brazil)	Atlantic Rain Forest
		#Brazil	
12	Ceratina (Ceratinula) sclerops Schrottky, 1907	* Encarnación (Itapúa, Paraguay)	Atlantic Rain Forest
		**Paraná, São Paulo (Brazil), Paraguay	
		#Rio Grande do Sul (Brazil)	
13	Ceratina (Ceratinula) turgida (Moure, 1941)	*Itatiaia (Rio de Janeiro, Brazil)	Atlantic Rain Forest
		#São Paulo, Paraná (Brazil)	
14	Ceratina (Ceratinula) xanthocera (Moure, 1941)	*Mar de Espanha (Minas Gerais, Brazil)	Atlantic Rain Forest

* Type Locality; ** Additional records from Moure Bee Catalog online version; #Additional records from Discover Life (including only those not mentioned in the Moure Bee Catalog online version).

These are solitary bees, whose role in the pollination of plants is still not well studied, although their importance in pollination of the melon cactus *Melocactus curvispinus* Pfeiff. and the carnivorous corkscrew plant *Genlisea violacea* A.St.-Hil. has been reported (Nassar and Ramírez 2004; Aranguren et al. 2018).

Information on the geographic distribution of the described species of C. (Ceratinula) is still limited. Moure (2012) did not mention any record for midwestern Brazil and recorded only four species for the northeastern region (*C. augochloroides*, *C. lucidula*, *C. manni*, and *C. muelleri* – Table 2). However, several local inventories record unidentified species in these regions (Sigrist et al. 2017 and Lima and Silvestre 2017 for midwestern Brazil; and Aguiar and Zanella 2005; Viana and Kleinert 2005; Albuquerque et al. 2007 and Milet-Pinheiro and Schlindwein 2007 for northeastern Brazil).

The present contribution describes and illustrates a distinctive new species of Ceratina (Ceratinula) Moure from midwestern Brazil (in the Cerrado Biome). Ceratina (C.) manni is redescribed, based on female and male individuals, and its geographical records updated. An identification key for the species of Ceratina (Ceratinula) so far recorded in Brazil is also provided, based on the list of Moure (2012).

## Materials and methods

The specimens of the new species described here were collected in November 2018, during a rapid assessment for monitoring bee diversity, performed in the area surrounding a soybean field on the Nossa Senhora Aparecida farm, in Água Fria de Goiás, Goiás State, midwestern Brazil. The farm belongs to the Fiorese family (Oli Antonio Fiorese, Edileusa Fiorese, Henrique Gustavo Fiorese, Kaio Felipe Fiorese), who have adapted their production methods to meet the standards for environmental certification, and this is now considered a model farm.

Repository institutions of the specimens are: Entomological Collection of the Natural History Museum (**MHNBA-MZUFBA**) of the Biology Institute of the Federal University of Bahia, Ondina Campus, Salvador, Bahia, Brazil; Entomological Collection of the Latin American Institute of Life and Nature Sciences of the Federal University of Latin American Integration (**CE-UNILA**), Foz do Iguaçu, Paraná, Brazil; Reference Collection of the Laboratory of Bionomy, Biogeography and Insect Systematics (**BIOSIS**), a unit associated with the MHNBA, in Salvador, Bahia, Brazil. The syntype of C. (Ceratinula) manni Cockerell, 1912 is deposited in the Entomological Collection of the American Museum of Natural History (**AMNH**), New York, United States of America.

The description of C. (Ceratinula) fioreseana sp. nov. is based on the female holotype and male paratypes. The redescription of the female of C. (Ceratinula) manni Cockerell, 1912 is based on all specimens studied, checking the diagnostic characters presented in the original description (Cockerell 1912) with images of the female syntype deposited in AMNH, which were downloaded from the Discover Life website in 2016 (Fig. 4 – the images are no longer available on the website).

Due to the COVID-19 pandemic, the identification key presented for Brazilian species of Ceratina (Ceratinula) was constructed based mainly on the original descriptions (as a first key attempt to aid identification, since the subgenus has not yet been revised), but was tested using a few specimens of seven different species that were accessible to us.

Specimens were studied and photographed using a Leica M165C stereomicroscope coupled to a Leica DFC295 digital camera, containing the program Leica Application Suite V4.1 Interactive Measurements, Montage. Measurements are given in millimeters and taken at the greatest width or length of structures. When we had access to more than one specimen, all were measured, and we report the average. The ocellocular distance is measured from the lateral ocellus, and the length of the anterior wing is measured from the costal sclerite. Abbreviations are:

**DO** diameter of the middle ocellus;

**DS** diameter of the scape;

**DP** diameter of a puncture;

**F1, F2, F3** antennal flagellomeres 1, 2 and 3;

**T** metasomal tergum;

**S** metasomal sternum.

The classification follows Michener (2007), whereby all bees correspond to the group Anthophila, and *Ceratina* is a genus within the family Apidae, subfamily Xylocopinae, tribe Ceratinini. Morphological characters in the identification key for recognition of Brazilian species follow their original descriptions and Moure’s (1941) redescriptions.

## Results

### *Ceratina* Latreille, 1802

#### Ceratina (Ceratinula)

Taxon classificationAnimaliaHymenopteraApidae

Moure, 1941

5BECC6B7-DA87-5FB5-8C20-D42B0485A719

##### Type species.

*Ceratina lucidula* Smith, 1854 by original designation.

##### Diagnosis.

Minute bees (3–6 mm long); usually metallic (rarely with the metasoma red); body elongated, with a slightly petiolate metasoma, the first segment as elongate-triangular; integument with extensive impunctate smooth areas, especially on head (paraocular area above antenna and on gena, sometimes on the whole head) and on mesonotum; second submarginal cell narrowed, sometimes converging to a point anteriorly, becoming almost triangular (Moure 1941; Michener 2007).

Among the species of Ceratina (Ceratinula) occurring in South America, C. (C.) fioreseana Oliveira, sp. nov. is similar to C. (C.) manni, especially in the pattern of facial maculation (Fig. 8). However, it is easily distinguished by the smaller and slimmer body, the color of the legs, antennal scape, pedicel and first three flagellomeres (honey-yellow in *fioreseana* sp. nov. and dark brown in *manni*); the integumental microsculpture on the paraocular region and on the upper part of the clypeus (smooth in *fioreseana* sp. nov. and microreticulate in *manni*); the yellow genal stripe (following the orbit in the lower region in *fioreseana* sp. nov. and on the upper portion of the head diverging upward from the orbit in *manni*).

The male genitalia also differ widely between the species, especially in the structure and shape of the valves, gonostyle, S5, S6 and S7 (Figs 3A–F; 7A–F).

#### Ceratina (Ceratinula) fioreseana

Taxon classificationAnimaliaHymenopteraApidae

Oliveira sp. nov.

F52FC9F9-CD3D-537C-84DC-36131D6FD584

http://zoobank.org/1EEDCDD0-999D-494E-ACC6-4EBCF775C031

**Figure 1.**
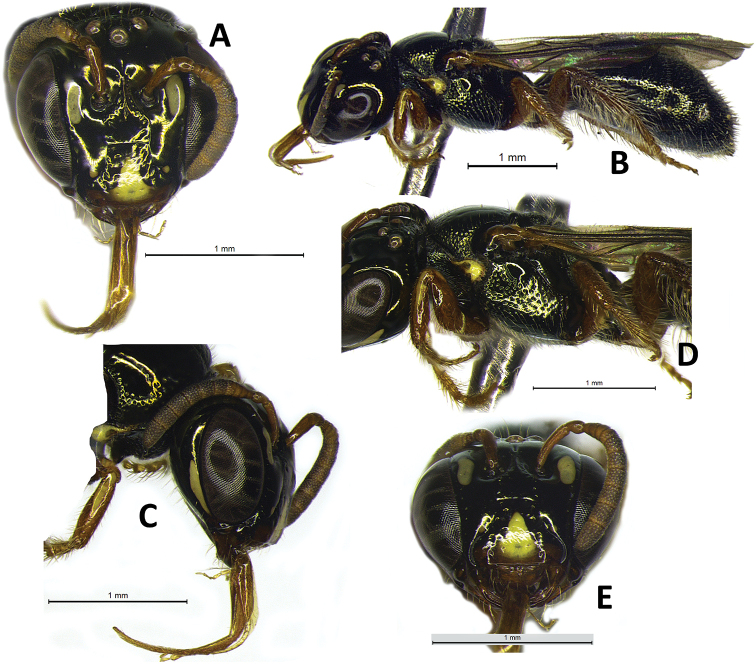
Female holotype of Ceratina (Ceratinula) fioreseana sp. nov., deposited at the Entomological Collection of the Natural History Museum of the Federal University of Bahia (MHNBA-MZUFBA), in Salvador, Bahia, Brazil **A** head in frontal view **B** body in lateral view **C** head in lateral view **D** mesosoma in lateral view **E** labrum in frontal view.

**Figure 2.**
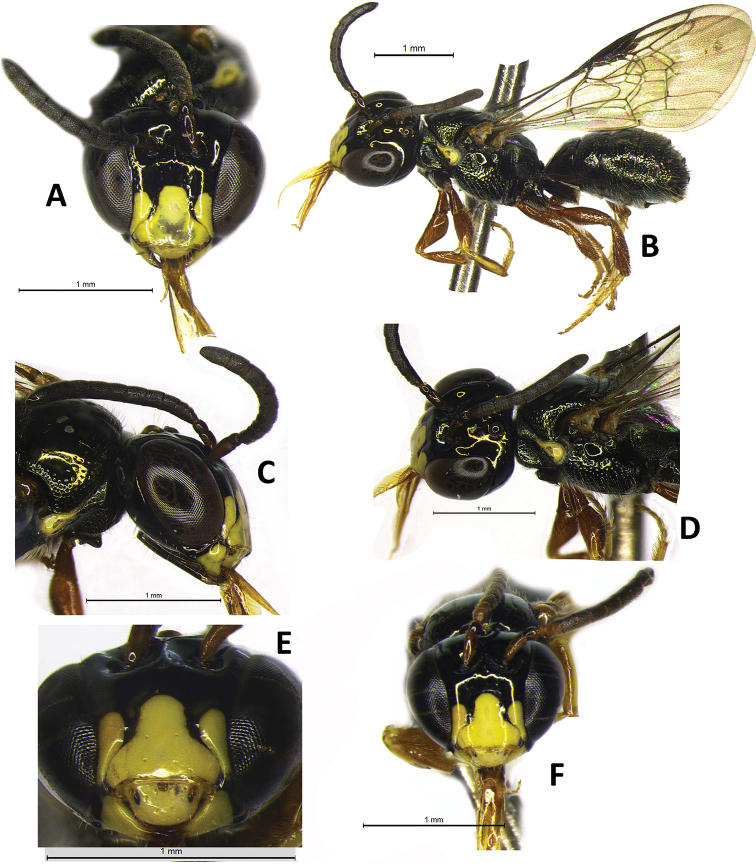
Male paratypes of Ceratina (Ceratinula) fioreseana sp. nov., deposited at the Entomological Collection of the Natural History Museum of the Federal University of Bahia (MHNBA-MZUFBA), in Salvador, Bahia, Brazil: Paratype **A** head in frontal view **B** body in lateral view **C** head in lateral view **D** mesosoma in lateral view. Paratype **E** labrum in frontal view **F** head in frontal view.

**Figure 3.**
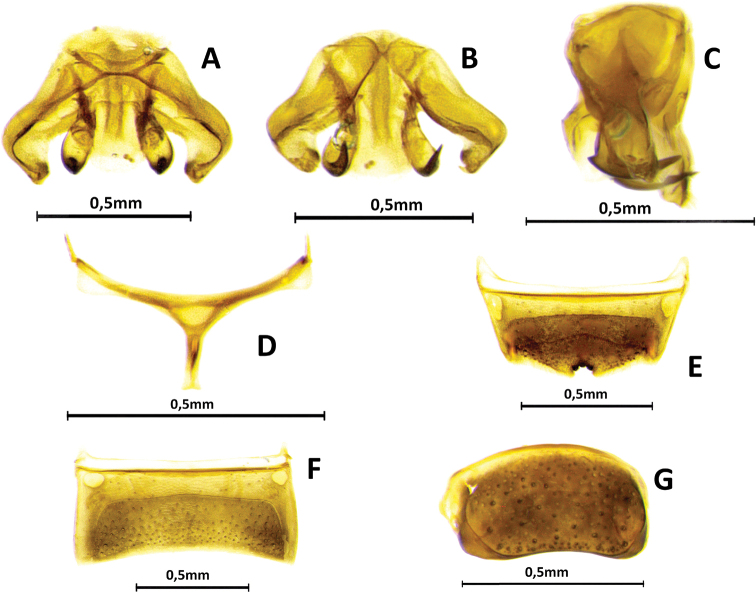
Male genitalia and associated metasomal sternum of paratype of Ceratina (Ceratinula) fioreseana sp. nov., deposited at the Entomological Collection of the Natural History Museum of the Federal University of Bahia (MHNBA-MZUFBA), in Salvador, Bahia, Brazil **A** genital capsule in dorsal view **B** genital capsule in ventral view **C** genital capsule in lateral view **D** seventh sternum in dorsal view (E7) **E** sixth sternum in dorsal view (E6) **F** fifth sternum in dorsal view (E5) **G** seventh tergum in dorso-lateral view.

Figures 1 , 2 , 3 , 8 , 9 , 10

##### Diagnosis.

**Both sexes**: integument color tending more to greenish with golden metallic sheen. **Female**: five yellow maculations on face and one stripe on gena; median paraocular yellow maculation not filling the entire space between the eye and antennal socket, and not reaching the height of upper part of the epistomal suture (Figs 1A, E; 8A; 9A, B); maculation of lower paraocular areas tiny, below tentorial pit (Figs 1A, E; 8A); lower paraocular area polished, smooth (Figs 1A; 8A); supraclypeal plain raised surface subpentagonal (Figs 1A; 8A; 9A, B); stripe of gena on lower half, adjacent to eye (Fig. 1C); antennal scape, pedicel and following three antennomeres honey-yellow (Fig. 1A, C, E); legs, from trochanters, honey-yellow (Fig. 1B–D). **Male**: clypeus almost totally yellow, except for a narrow strip that borders the upper edge above the tentorial pits; two large paraocular yellow spots close to clypeus; labrum and mandible almost entirely yellow; antennal scape and F1–F3 honey-yellow, pedicel brownish (Fig. 2A, B, F); apical margin of S5 uniformly concave (Fig. 3F); apical margin of S6 tri-concave, with deeper median concavity bearing three strongly sclerotized denticles (Fig. 3E); S7 narrow, less sclerotized, with very narrow base, concave on basal margin, with long narrow dorsally-directed, spatulate projection on median portion of apical margin (Fig. 3D).

##### Type locality.

Brazil, Goiás, Água Fria de Goiás, Fazenda Nossa Senhora Aparecida, Bayer Forward Farming, 14°49'25.946"S, 47°43'30.742"W (–14.823874, –47.725206), Cerrado vegetation (Savanna), alt. 1073 m a.s.l. (Fig. 10).

##### Description.

♀: ***Structure* (mm)**: total body length 4.9; forewing length 3.5; head width 1.37; eye length 0.83, width 0.47; gena width in profile 0.22; ocellocular distance 0.28; diameter of median ocellus 0.10; upper interorbital distance 0.86, median interorbital distance 0.75, lower interorbital distance 0.68; clypeus length 0.48, width 0.6; labrum length 0.17, width 0.36; scape length 0.3, width 0.08; F1 length 0.05; F2 length 0.05; F3 length 0.05; metatibia length 0.9, width 0.2; T2 width 1.25; T4 width 1.38. Antennal sockets located in deep depression (Fig. 8A); supraclypeal area level with clypeus and median paraocular region, the frons below; head sutures deep and distinct; a puncture line delimiting the supraclypeal plain raised area above, with lateral branches divergent basally, maximum diameter of puncture on line ca. 0.5 DS basally; supraclypeal plain raised surface subpentagonal (Figs 1A, C, E; 8A; 9A, B). ***Coloration***: integument mostly dark metallic golden-olive-green (Fig. 1 A–E), except for following parts: large elliptical longitudinal maculation on median paraocular area, extending upward and downward from level of antennal socket, not filling the entire space between the eye and antennal socket, and not reaching the height of upper part of the epistomal suture (maculation width ca. 1DS, length 2.3DS, ending at a height ca. 1.25DS from epistomal suture – scape maximum width – Figs 1A; 8A; 9A, B); large yellow subtriangular longitudinal maculation on disc of clypeus (Figs 1A, E; 8A); wide brownish honey-yellow band on apical 1/3 of clypeus, with base of subtriangular maculation on clypeus extending into this area (Fig. 1E); tiny round yellow maculation on lower paraocular area below tentorial pit (Fig. 1A, E); yellow stripe occupying lower half of gena, adjacent to eye (Fig. 1C); mandible reddish honey-brown, slightly darker on base and apex, with rounded translucent yellow area at base; labrum honey-yellow (Fig. 1E); antennal scape, pedicel and following three antennomeres honey-yellow (Fig. 1A, C, E); yellow maculation on pronotal lobe, outline areas translucent, reddish brown (Fig. 1B, D); legs honey-yellow from trochanter, meso- and meta- coxae slightly lighter brown, profemur with elliptical yellow maculation on apex of dorsal surface (Fig. 1B–D); light yellow longitudinal stripe on median line of dorsal surface of basal 1/2 of protibia (Fig. 1C); mesotibia with tiny pale-yellow spot on base of outer surface. ***Pubescence***: whitish, simple and sparse (Fig. 1B, D), shorter and sparser on head, denser on venter, longer on labrum (thicker), sides of mesosoma (especially on mesepisternum), metasoma (T3–T6) and legs, especially on metafemur and tibia; longest setae on face between ocelli (1.5DO, much finer), very short on clypeus, lower paraocular, supraclypeal and vertexal areas (0.75DO); gena nearly glabrous; sides of mesepisternum with pilosity relatively dense, sparse, long and uniformly distributed (1.5DO); posterior 2/3 of mesoscutum nearly glabrous; plumose setae easily visible only on pronotal lobe and surrounding areas (very short, whitish silvery), surrounding propodeal spiracle and on metatibia (ca. 3DO) (Fig. 1B, D); pilosity on metasoma simple, gradually longer and denser toward apex; denser on base and apical border of tergum, with glabrous area on disc from T1–T3 along its width; T4–T6 evenly setaceous; seta on sterna ca. 3DO. ***Microsculpture***: Integument smooth, polished, and shiny on most of surface; punctation piliferous, deep, distinct, and sparse. Punctures denser and more deep on sides of clypeus, upper part of supraclypeal area, anterior 1/3 of mesoscutum, mesepisternum and T4–T6, punctures larger on mesepisternum and mesoscutum and smaller on mesoscutellum and metanotum; metanotum and propodeum very coarsely microreticulate between sparse punctures; lower paraocular area, between antennal alveolus and tentorial pit, and near epistomal suture on upper half of clypeus, smooth, polished and shiny, not microreticulate; gena practically impunctate, smooth and polished; mesoscutum with punctation large, dense and deep on anterior 1/3, posterior 2/3 almost entirely smooth and polished, except for contours with small dense punctation; mesoscutellum with punctation very fine and dense, with impunctate polished area on disc; T1–T3 with punctation smaller and sparser, and broad glabrous impunctate polished area on each side of disc; T4–T6 with punctation evenly dense, large and deep.

♂: ***Structure* (mm)**: total body length 4.5; forewing length 3.5; head width 1.37; eye length 0.87, width 0.52; gena width in profile 0.17; ocellocular distance 0.24; diameter of median ocellus 0.12; upper interorbital distance 0.85, median interorbital distance 0.64, lower interorbital distance 0.64; clypeus length 0.42, width 0.49; labrum length 0.18, width 0.33; scape length 0.24, width 0.08; F1 length 0.05; F2 length 0.065; F3 length 0.07; metatibia length 0.83, width 0.18; T2 width 1.15; T4 width 1.38. Antennal sockets located in deep depression (Fig. 8B); frons and supraclypeal area level with clypeus and median paraocular region as in female but sutures less distinct (Fig. 2A, E, F); comparing with the female: eyes closer medially, scape shorter and wider, gena narrower in profile. ***Male terminalia***: apical margin of S5 uniformly concave (Fig. 3F); apical margin of E6 tri-concave, with deeper median concavity bearing three strongly sclerotized denticles (Fig. 3E); S7 narrow, less sclerotized, with very narrow base, concave on basal margin, with long narrow dorsally-directed spatulate projection on median portion of apical margin (Fig. 3D); gonostyle robust, enlarged and recurved, with an angulation in the middle portion almost forming 90 degrees, with apical portion directed to valves, lateral-distal surface of apical portion flattened, apex truncated (Fig. 3 A, B); valves in lateral view wider and subrectangular at base, hook-shaped in apical 1/4, with dentiform projection dorsomedially, which is connected to base by membranous/less-sclerotized transparent portion (Fig. 3A–C). ***Coloration***: similar to that of female, except for antennal pedicel brownish (Fig. 2A–F); clypeus yellow, narrowly black along epistomal suture from tentorial pit upward, apical margin yellowish brown, translucent (Figs 2A, E, F; 8B); labrum entirely yellow, with three rounded yellowish brown translucent maculations on each side and middle basically and apical contour equally yellowish brown, translucent (Fig. 2E); lower paraocular area yellow from slightly below base of clypeus downward, upper margin of maculation rounded (Figs 2A, E, F; 8B); mandible yellow, brownish red at base and apex (Fig. 2E); gena without yellow maculation (Fig. 2C); wide yellow stripe on external surface of protibia, from base to apex, occupying almost entire upper surface (Fig. 2B), tarsus pale yellow, contrasting with other segments of legs (Fig. 2B). ***Pubescence***: pilosity whitish as in female, slightly shorter and sparser, especially on mesoscutum, terga and legs. ***Microsculpture***: punctation finer and sparse, shallower, space between spots much larger (varying from 1–4DP), especially on mesoscutum, mesepisternum and terga; clypeus smooth, polished and shiny on most surface; larger smooth impunctate areas on T1–T3 and mesoscutellum.

##### Observed variations.

Some male specimens, such as the male paratype (Fig. 2A), have a discolored translucent area in the middle of the apical 1/2 of the clypeus. Other specimens have a uniformly yellow clypeus (Fig. 2F).

##### Type material.

(1♀, 3♂) – ***Holotype*** ♀ (MHNBA) // Brazil, Goiás, Água Fria de Goiás, Fazenda Nossa Senhora Aparecida, Bayer Forward Farming, 14°49'25.946"S, 47°43'30.742"W (–14.823874, –47.725206), 29.XI.2018, 15:00–15:34 h, Cerrado Savanna, alt. 1073 m a.s.l. ***Paratype used for description*** ♂ (MHNBA) // Brazil, Goiás, Água Fria de Goiás, Fazenda Nossa Senhora Aparecida, Bayer Forward Farming, 14°49'25.946"S, 47°43'30.742"W (–14.823874, –47.725206), 30.XI.2018, 10:40–11:40 h, Cerrado Savanna, alt. 1073 m a.s.l. ***Paratypes***: 2 ♂ (MHNBA) // Brazil, Goiás, Água Fria de Goiás, Fazenda Nossa Senhora Aparecida, Bayer Forward Farming, 14°49'25.946"S, 47°43'30.742"W (–14.823874, –47.725206), 29.XI.2018, 15:00–15:34 h, Cerrado Savanna, alt. 1073 m a.s.l. All specimens were collected with an entomological net on flowers of *Mentha piperita* L. (mint, family Lamiaceae), in planted vegetable garden.

##### Etymology.

The specific epithet is a patronym honoring Oli Antonio Fiorese, Edileusa Fiorese, Henrique Gustavo Fiorese and Kaio Felipe Fiorese, owners of the Nossa Senhora Aparecida farm (located in Água Fria de Goiás, Goiás State, midwestern Brazil), where the type specimens of the new species were collected. We honor their recognition of bees as key pollinators important to sustainable production, adapting their production to meet the standards for environmental certification, and also adopting various pollinator-friendly measures through the Bayer Forward Farming Project. Because of these procedures, their property has been certified as a model farm by environmental agency Round Table on Responsible Soy Association (RTRS), being the only farm in Brazil within the Bayer Forward Farming Project, and the twenty-fifth in the world.

#### Ceratina (Ceratinula) manni

Taxon classificationAnimaliaHymenopteraApidae

Cockerell, 1912

FD4FED6E-8E01-59BA-AE69-985717F57ABC

**Figure 4.**
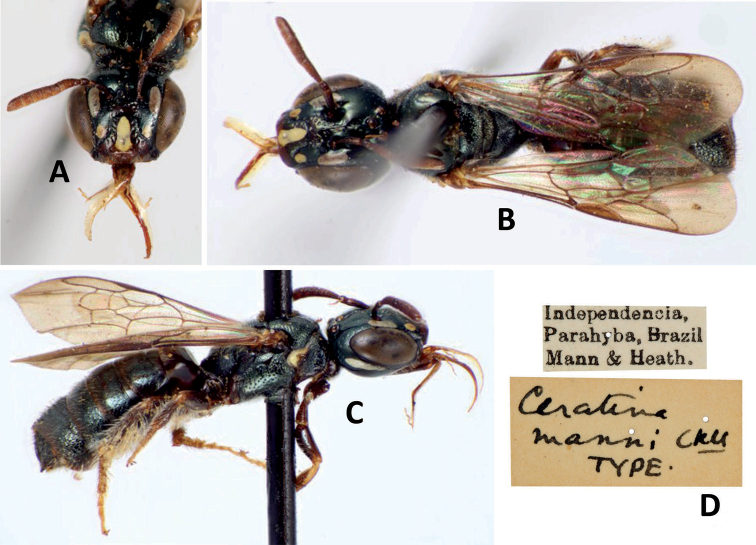
Female syntype of Ceratina (Ceratinula) manni Cockerell, 1912 deposited at the Entomological Collection of the American Museum of Natural History (AMNH, New York, United States of America) **A** head in frontal view (Photograph: I_HHG3587) **B** body in dorsal view (Photograph: I_HHG3585) **C** body in lateral view (Photograph: I_HHG3586) **D** labels (Photograph: I_HHG3588). Ownership rights to these images and their copyright belong to AMNH and Hadel Go. Photographs by Copyright Hadel Go 2011-2016 downloaded from the Discover Life Website.

**Figure 5.**
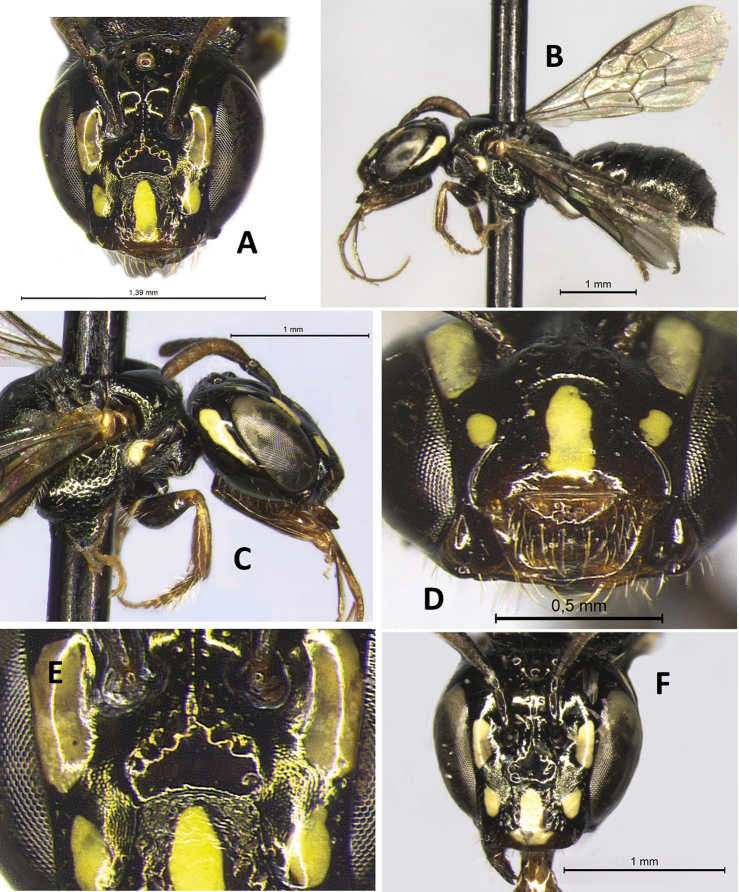
Female of Ceratina (Ceratinula) manni Cockerell, 1912 **A** head in frontal view **B** body in lateral view **C** head and mesosoma in lateral view **D** labrum in frontal view **E** median paraocular area and clypeus in detail **F** head in frontal view **A, E** specimen from Amélia Rodrigues, Bahia, deposited at the Reference Collection of the Laboratório de Bionomia, Biogeografia e Sistemática de Insetos (BIOSIS), Federal University of Bahia (MHNBA-MZUFBA), in Salvador, Bahia, Brazil; Specimens **B,C, F** from Caracol, Piauí, and **D** Milagres, Bahia, deposited at the Entomological Collection of the Federal University of Integração Latino-Americana (CE-UNILA), in Foz do Iguaçu, Paraná, Brazil.

**Figure 6.**
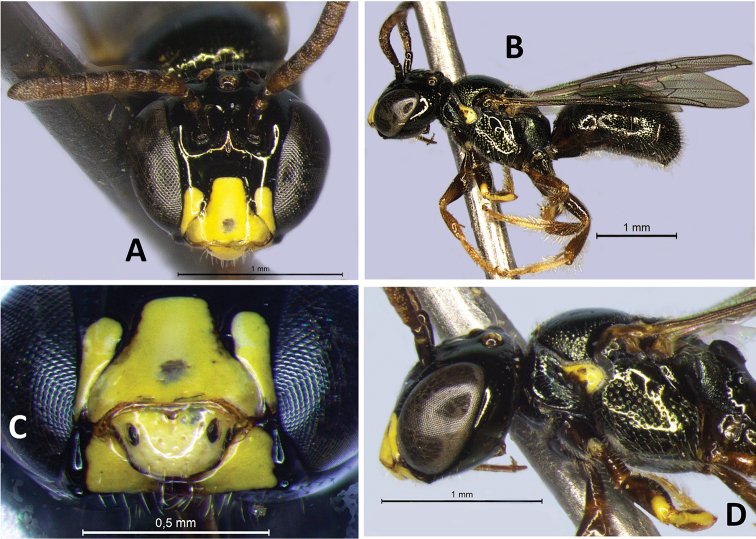
Male of Ceratina (Ceratinula) manni Cockerell, 1912, specimen from Crato, Ceará, deposited at the Entomological Collection of the Federal University of Integração Latino-Americana (CE-UNILA), in Foz do Iguaçu, Paraná, Brazil **A** head in frontal view **B** body in lateral view **C** labrum in frontal view **D** head and mesosoma in lateral view.

**Figure 7.**
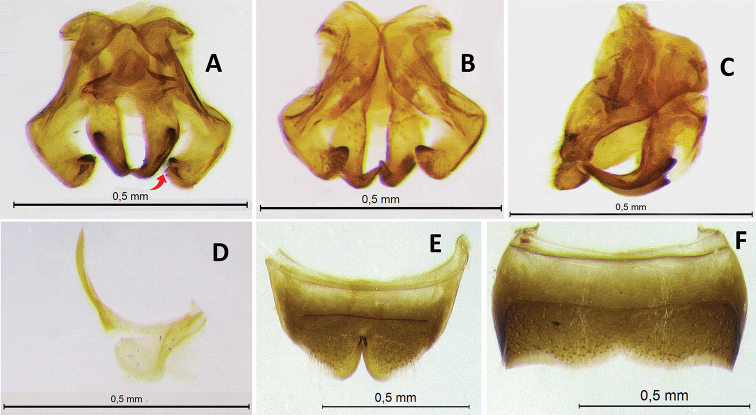
Male genitalia and associated metasomal sternum of Ceratina (Ceratinula) manni Cockerell, 1912, specimen from Serra Negra do Norte, Rio Grande do Norte, Brazil, deposited at the Entomological Collection of the Federal University of Integração Latino-Americana (CE-UNILA), in Foz do Iguaçu, Paraná, Brazil **A** genital capsule in dorsal view (red arrow shows the bidentate apex) **B** genital capsule in ventral view **C** genital capsule in lateral view **D** seventh sternum in dorsal view (E7) **E** sixth sternum in dorsal view (E6) **F** fifth sternum in dorsal view (E5).

**Figure 8.**
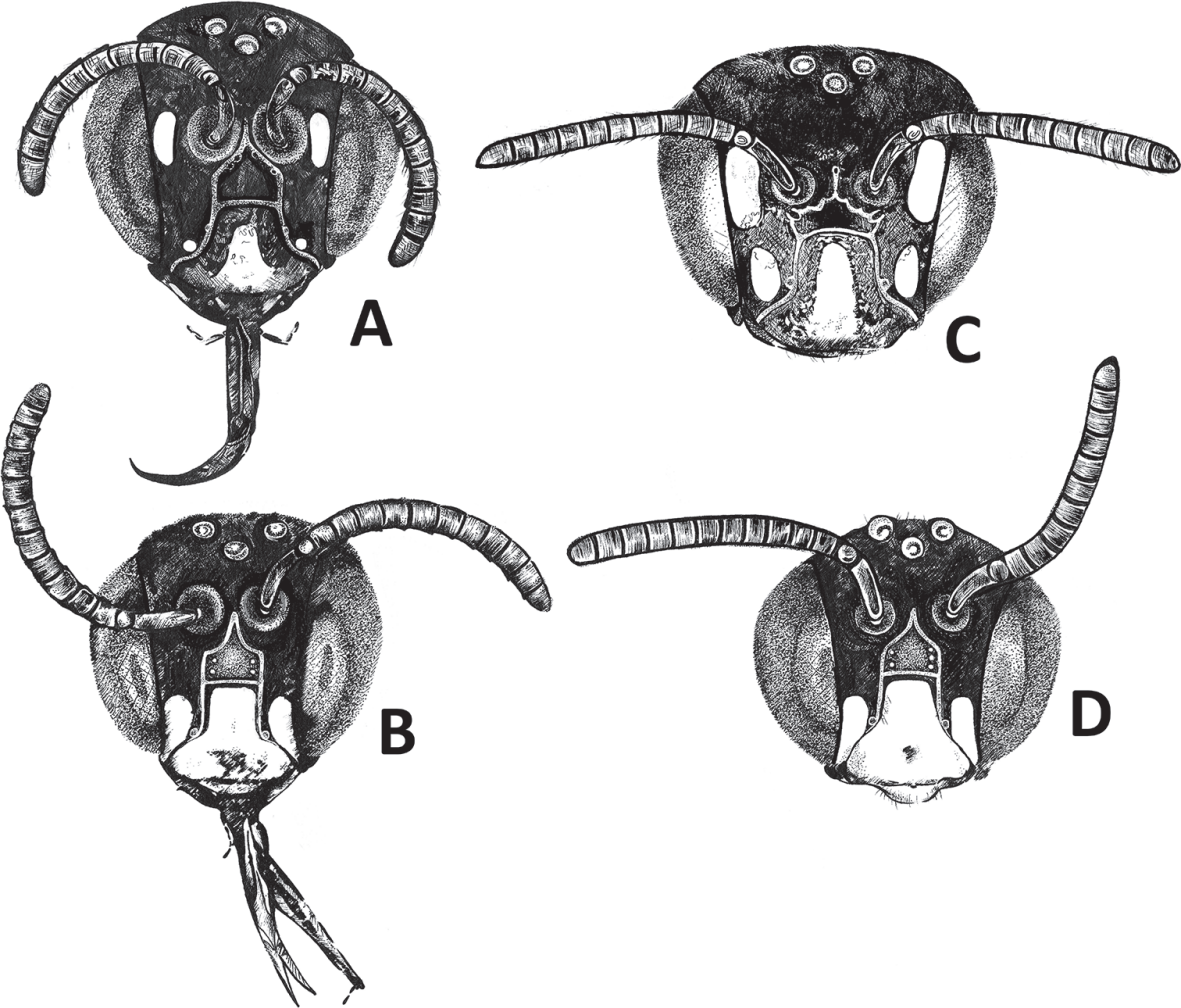
Head of Ceratina (Ceratinula) Moure, 1941 species in frontal view **A** (female holotype) **B** (male paratype) Specimens of Ceratina (Ceratinula) fioreseana sp. nov., deposited at the Entomological Collection of the Natural History Museum of the Federal University of Bahia (MHNBA-MZUFBA), in Salvador, Bahia, Brazil **C, D**Ceratina (Ceratinula) manni Cockerell, 1912: **C** female specimen from Amélia Rodrigues, Bahia, deposited at the Reference Collection of the Laboratório de Bionomia, Biogeografia e Sistemática de Insetos (BIOSIS), Federal University of Bahia (MHNBA-MZUFBA), in Salvador, Bahia, Brazil **D** male specimen from Crato, Ceará, deposited at the Entomological Collection of the Federal University of Integração Latino-Americana (CE-UNILA), in Foz do Iguaçu, Paraná, Brazil. Drawings by Luisa de Lima Ruschioni.

**Figure 9.**
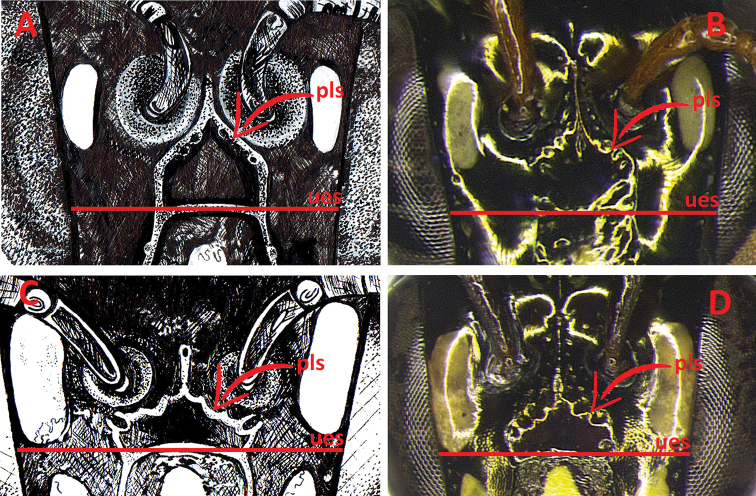
Head of Ceratina (Ceratinula) Moure, 1941 species in frontal view, showing the supraclypeal area and median paraocular area **A, B** female holotype of Ceratina (Ceratinula) fioreseana sp. nov., specimen deposited at the Entomological Collection of the Natural History Museum of the Federal University of Bahia (MHNBA-MZUFBA), in Salvador, Bahia, Brazil **C, D** female specimen of Ceratina (Ceratinula) manni Cockerell, 1912 from Amélia Rodrigues, Bahia, deposited at the Reference Collection of the Laboratório de Bionomia, Biogeografia e Sistemática de Insetos (BIOSIS), Federal University of Bahia (MHNBA-MZUFBA), in Salvador, Bahia, Brazil. Details: pls = puncture line delimiting the supraclypeal plain raised area above; ues = upper part of the epistomal suture.

**Figure 10.**
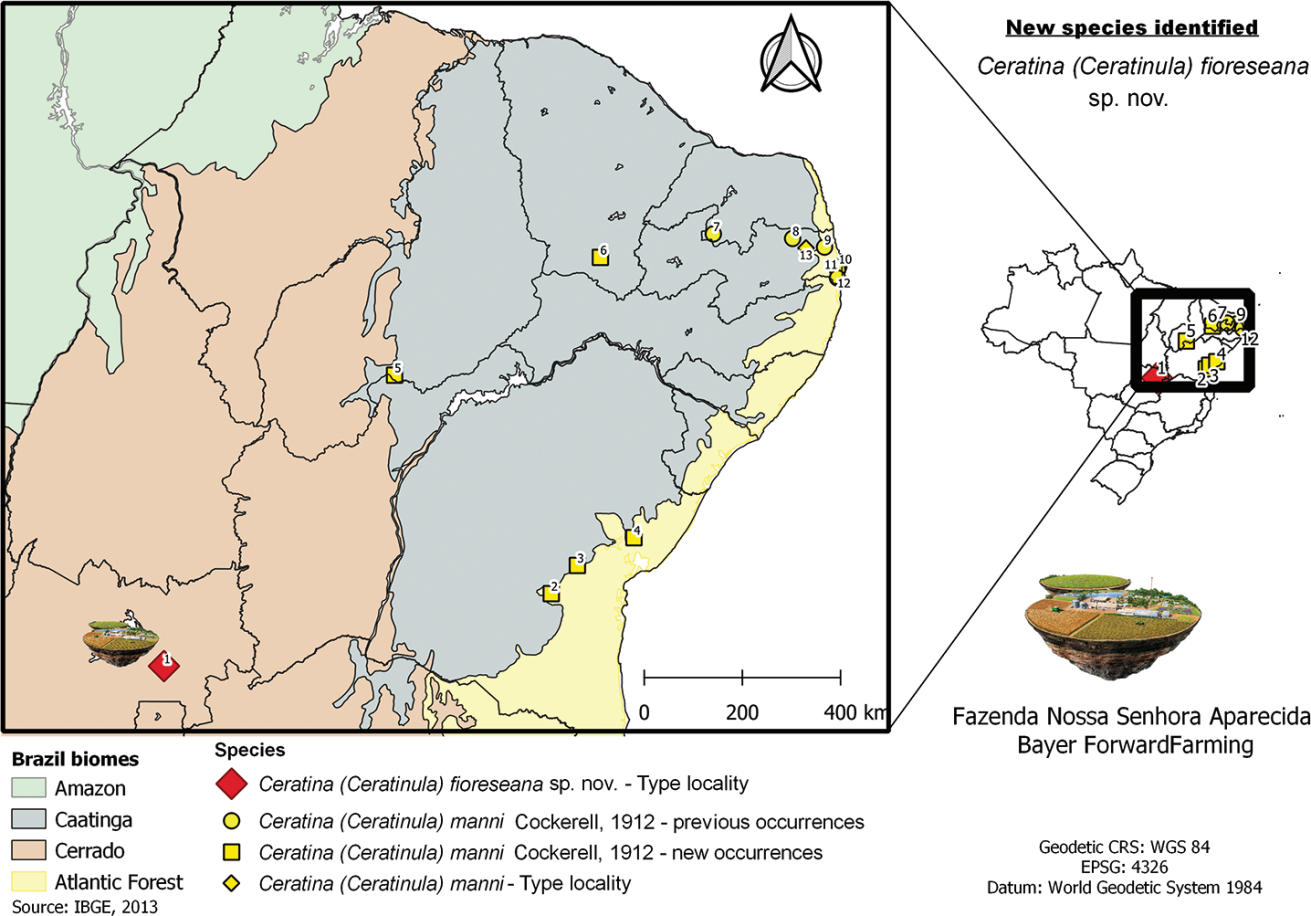
Geographic distribution map of Ceratina (Ceratinula) fioreseana sp. nov. and Ceratina (Ceratinula) manni Cockerell, 1912 in and Brazil: (1) Goiás, Água Fria de Goiás, Fazenda Nossa Senhora Aparecida, Bayer Forward Farming (*fioreseana* Type locality); (2) Bahia, Maracás; (3) Bahia, Milagres; (4) Bahia, Amélia Rodrigues; (5) Piauí, Caracol; (6) Ceará, Crato; (7) Rio Grande do Norte, Serra Negra do Norte, Estação Ecológica do Seridó; (8) Paraíba, Cacimba de Dentro, Fazenda Cachoeira da Capivara; (9) Paraíba, Mamanguape; (10) Paraiba, João Pessoa, Periurban Area – Sítio ponta de Gramame; (11) Paraíba, Conde, Granja Pitumirim; (12) Paraíba, Alhandra, Assentamento Tapuiu – Sítio Olho D’água; (13) Paraíba, Guarabira (*manni* Type locality).

Figures 4 , 5 , 6 , 7 , 8 , 9 , 10

Ceratina manni Cockerell, 1912: 47 [original description]; Schwarz 1943:30 [citation]; Michener 1954: 152 [citation]; ITS, 2009 [online catalog, geographic distribution]; Discover Life 2020 [online catalog, geographic distribution].
Ceratinula manni : Zanella 2000: 591 [biogeography]; Zanella 2003: 235 [biogeography];
Ceratina (Ceratinula) manni : Silveira et al. 2002: 146 [*partim*; geographic distribution]; Aguiar and Martins 2003: 213 [biogeography]; Zanella and Martins 2005: 383 [biogeography]; Moure et al. 2012 [online catalog, geographic distribution]; Cruz 2013 [floral visit record].

##### Type locality.

Brazil, Paraíba, Guarabira (previously known as Independencia).

##### Diagnosis.

**Both sexes**: integument color tending more to greenish with golden metallic sheen. **Female**: five yellow maculations on face and one stripe on gena; median paraocular yellow maculation almost filling the entire space between the eye and antennal socket, and almost reaching the height of upper part of the epistomal suture (Fig. 9, C, D); oval maculation on lower paraocular areas large, near tentorial pit (Figs 4A, B; 5A, D–F; 8C); lower paraocular area microreticulate (Figs 5E; 8C); supraclypeal plain raised surface subtriangular (Fig. 9, A, B); stripe of gena on superior half, extending above dorsal margin of eye, broader and divergent superiorly and closer to eye in lower portion (Figs 4C; 5B, C); antennal scape, pedicel and following three antennomeres brown (Figs 4A–C; 5A–F); coxae, trochanters and femurs of all legs brown, protibia and tarsus lighter honey-brown, meso- and metatibiae and basitarsi lighter brown, following tarsomeres lighter honey-brown (Figs 4C; 5B, C). **Male**: clypeus almost totally yellow, except for a narrow strip that borders the upper edge above the tentorial pits; two large paraocular yellow spots close to clypeus; labrum and mandible almost entirely yellow; apical margin of S5 slightly tri-concave, median concavity deepest (Fig. 7F); apical margin of S6 strongly bilobed, with deep median concavity intruding almost to midlength of sternum (Fig. 7E).

##### Description.

♀: ***Structure* (mm)**: total body length 4.7; forewing length 3.2; head width 1.3; eye length 0.89, width 0.43; gena width in profile 0.26; ocellocular distance 0.33; diameter of median ocellus 0.12; upper interorbital distance 0.91, median interorbital distance 0.78, lower interorbital distance 0.67; clypeus length 0.43, width 0.61; labrum length 0.22, width 0.44; scape length 0.31, width 0.09; F1 length 0.09; F2 length 0.05; F3 length 0.05; metatibia length 0.54, width 0.16; T2 width 1.25; T4 width 1.45. Antennal sockets located in shallow depression (Fig. 8C), frons and supraclypeal area raised above clypeus and median paraocular region, head sutures shallow; a puncture line delimiting the supraclypeal plain raised area above, with lateral branches divergent basally, maximum diameter of puncture on line ca. 1 DS basally; supraclypeal plain raised surface subtriangular (Figs 5A, E, F; 8C; 9C, D). ***Coloration***: integument mostly dark metallic golden-olive-green (Figs 4A–C; 5A–F), except following parts: large elliptical longitudinal yellow maculation in median paraocular area, extending upward and downward from level of antennal socket, almost filling the entire space between the eye and antennal socket, and almost reaching the height of upper part of the epistomal suture (maculation width ca. 1.4DS, length 2.4DS, ending at a height ca. 0.5DS – scape maximum width – Figs 5A, E, F; 8C; 9C, D); large yellow subtriangular longitudinal maculation on disc of clypeus (Figs 4A, B; 5A, D, E, F; 8C); oval relatively large yellow maculation on lower paraocular areas near tentorial pit (Figs 4A, B; 5A, D, E, F; 8C); wide brownish honey-yellow band on apical 1/3 of clypeus (Fig. 4A, B); yellow stripe occupying superior half of gena, extending above dorsal margin of eye, broader and divergent superiorly and closer to eye in lower portion (Figs 4C; 5B, C); mandible honey-brown, more reddish on base and more blackened on apex; labrum honey-brown, slightly lighter basally (Fig. 5D); antennal scape, pedicel and first three flagellomeres brown, scape with tiny dark honey-brown area on basis and apex (Figs 4A–C; 5A, C, F); yellow maculation on pronotal lobe demarcated by translucent reddish brown band (Figs 4C; 5B, C); coxae, trochanters and femurs of all legs brown with slight dark-olive-green metallic sheen; profemur with lighter-brown apical area; protibia and tarsus lighter honey-brown, tibia with longitudinal yellow stripe dorsally in basal 1/2; meso- and metatibiae and basitarsi lighter brown, following tarsomeres honey-brown; meso- and metatibiae with tiny pale-yellow spot on base of dorsal surface (Figs 4C; 5B, C). ***Pubescence***: whitish, simple and sparse, shorter and sparser on head, denser on venter, longer on labrum (very coarse), sides of mesosoma, metasoma (T3–T6) and legs, especially on metafemur and tibia; longest setae on face between ocelli (1.5DO, much finer), very short on clypeus, lower paraocular, supraclypeal, and vertexal areas (0.5DO); gena nearly glabrous; sides of mesepisternum with relatively dense, long, uniformly distributed pilosity (1.5DO); posterior 2/3 of mesoscutum nearly glabrous; plumose setae easily visible only on pronotal lobe and its surroundings (very short, whitish silver), surrounding propodeal spiracle and on metatibia (ca. 3DO); pilosity on metasoma simple, gradually longer and denser toward apex; denser on base and apical border of tergum; T1–T3 with glabrous area on disc; T4–T6 evenly setaceous; setae on sterna ca. 2.25DO. ***Microsculpture***: Integument impunctate, polished and shiny on most of surface; punctation piliferous, deep and sparse. Punctures denser and deeper on supraclypeal area, anterior 1/3 of mesoscutum, mesepisternum and T4–T6, punctures larger on face and smaller on mesoscutellum; metanotum and propodeum very coarsely microreticulate between sparse punctures; finely microreticulate area on lower paraocular area, between antennal alveolus and tentorial pit, also near epistomal suture on upper half of clypeus (Fig. 5E); gena nearly impunctate, smooth and polished with some very superficial large punctures in middle longitudinally on yellow stripe and some denser and deeper punctures in upper portion (Fig. 5C); mesoscutum with punctation large, dense and deep on anterior 1/3, posterior 2/3 nearly smooth and polished, except for contours with dense small punctures; mesoscutellum with punctation very fine and dense, and smooth polished area on each side of disc; T1–T3 with punctation very fine and sparse, and broad glabrous smooth polished area on each side of disc; T4–T6 with punctation evenly dense, coarse and marked.

♂: ***Structure* (mm)**: total body length 3.9; forewing length 3.06; head width 1.3; eye length 0.76, width 0.46; gena width in profile 0.23; ocellocular distance 0.24; diameter of median ocellus 0.12; upper interorbital distance 0.84, medium interorbital distance 0.61, lower interorbital distance 0.57; clypeus length 0.49, width 0.57; labrum length 0.21, width 0.33; scape length 0.23, width 0.08; F1 length 0.05; F2 length 0.04; F3 length 0.06; metatibia length 0.5, width 0.14; metasomal width 1.19 (measured on T4). Antennal sockets located in shallow depression (Fig. 8D), frons and supraclypeal area raised above clypeus and median paraocular area, head sutures shallow (Fig. 6A); comparing with the female: eyes closer medially, scape shorter and wider, gena narrower in profile (Fig. 6D). ***Male terminalia***: apical margin of S5 slightly tri-concave, median concavity deepest (Fig. 7F); apical margin of S6 strongly bilobed, with deep median concavity intruding almost to midlength of sternum (Fig. 7E); S7 quite narrow and less sclerotized, almost transparent in median portion which is wider, apical margin rounded (Fig. 7D; this structure was broken, with part missing, and not possible to see in its entirety); gonostyle robust, enlarged and recurved, with an angulation in the median and preapical portion almost forming a 90 degrees, lateral-distal surface flattened, apical portion directed to valves ending in narrow bidentate apex (Fig. 7A); valves slender in apical 1/2, hook-shaped, with dentiform projection dorsomedially, which is connected to base by less-sclerotized, transparent membranous portion (Fig. 7A–C). ***Coloration***: similar to that of female (Fig. 6A–D) except clypeus yellow, narrowly black along epistomal suture from tentorial pit upward, honey-brown translucent stripe on apical border and darker irregularly rounded honey-brown translucent maculation in middle of disc (Fig. 6A, C); labrum yellow, paler than clypeus, with paired oval translucent brown maculation laterally, and apical margin translucent brown (Fig. 6C); lower paraocular area yellow from slightly below base of clypeus downward, upper margin of maculation rounded (Figs 6A, C, 8D); mandible yellow, brownish at base and apex (Fig. 6C); gena without yellow stripe (Fig. 6B, D); apex of scape lighter brown; legs with small yellow maculation on apices of femurs and base of tibiae; wide yellow stripe on protibia, from base to apex, occupying almost entire dorsal surface (Fig. 6B, D); tarsus entirely yellow (Fig. 6B). ***Pubescence***: pilosity whitish as in female, slightly shorter and sparse, especially on mesoscutum, terga and legs. ***Microsculpture***: punctures smaller and sparser, interspaces much larger, especially on mesoscutum, mesepisternum and tergum; clypeus smooth, polished and shiny on most surface; with smooth impunctate areas on T1–T3 slightly larger, as well as those of mesoscutellum; microreticulation of metanotum and basal area of propodeum shallower.

##### Observed variations.

In females, the large elliptical yellow longitudinal maculation on the disc of the clypeus is sometimes enlarged apically, as observed in some specimens from Piauí State; these also have a small translucent brown oval maculation in the middle of the disc (Fig. 5F), invading the area of the wide brownish honey-yellow band on the apical third of the clypeus.

##### Distribution

(new geographical records indicated by*). *Ceratina manni* is endemic to northeastern Brazil and occurs mainly within the limits of the Caatinga region (Fig. 10), being recorded from near sea level to 945 m a.s.l. The records from João Pessoa and vicinity by Cruz (2013) in peri-urban and rural areas and in Mamanguape, all in Paraíba, indicate that the species is not restricted to the semiarid region, and occurs in other areas of open vegetation, at least near the limit of the Caatinga (open dry diagonal, or South American diagonal of open formations – Vanzolini 1974). The record from São Paulo state (Salesópolis, Boracéia Biological Station – Wilms 1995), secondarily cited by Pedro and Camargo (2000), Imperatriz-Fonseca et al. (2011) and in the Discover Life website (2020 -Table 2) must be checked (see remarks below).

**Brazil**: *Piauí State: Caracol. *Ceará state: Crato. **Rio Grande do Norte state**: Serra Negra do Norte (Zanella 2000, 2003). **Paraíba state**: Cacimba de Dentro (Zanella and Martins 2005), Alhambra, Conde, João Pessoa (Cruz 2013), Mamanguape (Aguiar and Martins 2003). *Bahia state: Amélia Rodrigues, Maracás, Milagres.

##### Material examined

(11♀, 2♂). 1 ♂ // (CE-UNILA HYAP 6072) // Brasil, Rio Grande do Norte, Serra Negra do Norte, Estação Ecológica do Seridó, 17.xii.1994, Zanella FCV and Moura ON *leg.*; 1 ♀ // (CE-UNILA HYAP 6073) // Brasil, Rio Grande do Norte, Serra Negra do Norte, Estação Ecológica do Seridó, 30.xii.1994, Zanella FCV and Moura ON *leg.*; 1 ♀ // (CE-UNILA HYAP 3350) // Brasil, Paraíba, Cacimba de Dentro, Fazenda Cachoeira da Capivara, 25.x.2003, F. Zanella *leg.*; 1 ♀ // (CE-UNILA HYAP 1109) // Brasil, Piauí, Caracol, 518 m, 13.xii.2010, F. Zanella and A. Carvalho *leg.*; 1 ♀ // (CE-UNILA HYAP 1110) // Brasil, Piauí, Caracol, 518 m, 13.xii.2010, F. Zanella and A. Carvalho *leg.*; 1 ♂ // (CE-UNILA HYAP 1120) // Brasil, Ceará, Crato, Estrada para Exú, Encosta, 07.ii.2011, F. Zanella *leg.*; 1 ♀ // (CE-UNILA HYAP 1117) // Brasil, Bahia, Maracás, 13°26'33.8"S, 40°20'42.6"W, 945 m, 13.iii.2012, Zanella FCV. *leg.*; 1 ♀ // (CE-UNILA HYAP 1118) // Brasil, Bahia, Maracás, 13°26'33.8"S, 40°20'42.6"W, 945 m, 13.iii.2012, Zanella FCV. *leg.*; 1 ♀ // (CE-UNILA HYAP 1112) // Brasil, Bahia, Milagres, 12°54'19.2"S, 39°50'46.5"W, 758 m, 16.iii.2012, Zanella FCV. *leg.*; 1 ♀ // (CE-UNILA HYAP 1115) // Brasil, Bahia, Milagres, 12°54'19.2"S, 39°50'46.5"W, 758 m, 16.iii.2012, Zanella FCV. *leg.*; 1 ♀ // (BIOSIS-UFBA, Favízia 06380) // Brasil, Bahia, Amélia Rodrigues, 12°22'31.70"S, 38°46'05.82"W, 21.i.2017, Hora: 13:10 P. 11, n° 404, on flowers of *Stemodia foliosa* Benth. Silva, Anjos and Melo *leg.*; 1 ♀ // (BIOSIS-UFBA, Favízia 06381) // Brasil, Bahia, Amélia Rodrigues, 12°22'31.70"S, 38°46'5.82"W, 21.i.2017, Hora: 13:10 P. 11, n° 405, on flowers of *Stemodia foliosa* Benth. Silva, Anjos and Melo *leg.*; 1 ♀ // (BIOSIS-UFBA, Favízia 06382) // Brasil, Bahia, Amélia Rodrigues, 12°22'31.70"S, 38°46'5.82"W, 11.iii.2017, Hora: 10:00 P. 11, on flowers of *Stemodia foliosa* Benth. Silva, Anjos and Melo *leg.* (Fig. 10).

##### Remarks.

Wilms (1995:52) reported *C. manni* from the Boracéia Biological Station (2 females), located in Salesópolis, São Paulo, southeastern Brazil. These specimens would have been deposited in the Zoological Museum of São Paulo University (MZSP) and examined by Pedro and Camargo (2000). Unfortunately, the material collected by Wilms (1995) and cited by Pedro and Camargo (2000), Silveira et al. (2002), Imperatriz-Fonseca et al. (2011), and in the Discover Life website (2020 – Table 2) could not be located, so this record must be verified. Additionally, this record from Boraceia is from the Atlantic Forest in southeastern Brazil, far from the Caatinga, at an altitude of approximately 800 m a.s.l. and in a tropical rain forest with more than 3000 mm mean annual rainfall (Wilms 1995).

#### Identification key for females of Ceratina (Ceratinula) Moure, 1941

Species so far recorded for Brazil, according to the “Catalogue of Bees (Hymenoptera, Apoidea) in the Neotropical Region” (Moure 2012).

The identification key presented above is probably not exhaustive, as there are likely to be many species that have not yet been described or recorded in Brazil, but are known from neighboring countries. Therefore, we recommend that the characters be verified in the original descriptions and with a reference collection or type material, in order to more confidently assign species names.

**Table:** 

1	Face with yellow-pigmented maculations	**2**
–	Face without yellow-pigmented maculations	**11**
2	Yellow maculations present on clypeus and on paraocular region; with yellow genal stripe; yellowish or whitish pronotal lobe	**3**
–	Yellow maculations restricted to either clypeus or to paraocular region, if on both, then only in lower paraocular area; lacking yellow genal stripe; pronotal lobe variable	**5**
3	Color tending more to greenish with golden metallic sheen; scape color variable. Facial pattern consisting of five yellow maculations: one large elliptical longitudinal maculation in median paraocular area, extending upward and downward from level of antennal socket, almost reaching or not the height of upper part of the epistomal suture; one smaller rounded maculation on lower paraocular area near tentorial pit; one median, longitudinal subtriangular maculation occupying large area of clypeus; dorsal face of supraclypeal raised surface subtriangular or subpentagonal; frons level variable	**4**
–	Color tending more to brownish, with strong blue-violet sheen on head, mesosutum an metasoma; scape reddish brown. Facial pattern consisting of three yellow maculations: one large elliptical longitudinal maculation in median paraocular area, extending upward and downward from level of antennal socket, not reaching the height of upper part of the epistomal suture (maculation width ca. 1.5DS, length 4DS, ending at a height ca. 1.2DS from epistomal suture – scape maximum width); and one median, longitudinal subtriangular maculation occupying large area of clypeus; supraclypeal plain raised surface subrectangular; frons below the rest of the head	**Ceratina (Ceratinula) muelleri Friese, 1910** (Brazilian States: Amazonas, Pará, Maranhão, Ceará, Minas Gerais, Rio de Janeiro, São Paulo, Paraná; Argentina)^1^
4	Yellow maculation on median paraocular area almost reaching the height of upper part of the epistomal suture (maculation width ca. 1.4DS, length 2.4DS, ending at a height ca. 0.5DS from epistomal suture – scape maximum width – Figs 5A, E, F; 8C; 9C, D); yellow maculations on lower paraocular area large, oval, near tentorial pit (Fig. 8C); scape, pedicel and first three flagellomeres dark blackish brown (Fig. 5A); upper corners of clypeus and adjacent paraocular region with microreticulation clearly visible (Figs 5E; 8C); protibia and tarsus yellow-honey; trochanters, femurs and tibiae of meso- and metalegs blackened (Fig. 5B); genal yellow stripe located in upper region of gena, diverging from orbit in upper portion (Fig. 5C); antennal sockets located in shallow depression, frons and supraclypeal area raised above clypeus and median paraocular region (Fig. 5A, E), head sutures shallow; supraclypeal plain raised surface subtriangular (Fig. 9A, B)	**Ceratina (Ceratinula) manni Cockerell, 1912** (Brazilian States: Piauí, Ceará, Rio Grande do Norte, Paraíba, Bahia – Caatinga biome and ecotone zone)
–	Yellow maculation on median paraocular area not reaching the height of upper part of the epistomal suture (maculation width ca. 1DS, length 2.3DS, ending at a height ca. 1.25DS from epistomal suture – scape maximum width – Figs 1A; 8A; 9A, B); yellow maculations on lower paraocular area smaller, rounded, below tentorial pit (Fig. 8A); scape, pedicel and first three flagellomeres yellow-honey; upper corners of clypeus and adjacent paraocular area with smooth polished surfaces (Figs 1A; 8A); pro, meso and metalegs completely honey-yellow (Fig. 1B); genal stripe located in lower genal region, adjacent to orbit (Fig. 1C); antennal sockets located in deep depression; supraclypeal area level with clypeus and median paraocular region (1A), the frons below, head sutures deep; supraclypeal plain raised surface subpentagonal (Figs 1A; 8A; 9A, B)	**Ceratina (Ceratinula) fioreseana Oliveira, sp. nov.** (Brazil: Goiás State – Cerrado biome)
5	Facial maculations restricted to median paraocular region, close to antennae	**6**
–	Facial maculations restricted to lower paraocular region and clypeus	**8**
6	Larger in size (5 mm to larger); small facial maculations, width smaller than scape diameter	**7**
–	Smaller in size (4–4,5 mm); large facial maculations, width larger than scape diameter	**Ceratina (Ceratinula) biguttulata (Moure, 1941)** (Brazil: Paraná State)
7	Yellow maculations very small, almost imperceptible; anterior part of scape and meso and metatarsi, yellowish brown; very sparse punctation (3–4DP); metatibia strongly thickened	**Ceratina (Ceratinula) melanochroa (Moure, 1941)** (Brazil: Paraná State)
–	Yellow maculations small, but distinct, elongated; antennae dark brown with underside of flagellum rust-brown; meso and metatarsi dark brown; punctation less sparse (2–3DP); metatibia normal	**Ceratina (Ceratinula) sclerops Schrottky, 1907** (Brazilian states: São Paulo, Paraná; Paraguay)
8	Variable in size; antennal scape and meso and metatibiae blackened; other characters, variable	**9**
–	Small in size (around 4 mm); antennal scape, tibiae and tarsus, tegulae and wing venation honey-yellow; small elongated maculation on clypeus; pronotal lobes with yellow maculation	**Ceratina (Ceratinula) xanthocera (Moure, 1941)** (Brazil: Minas Gerais State)
9	Normal face, with shallow sutures; head nearly glabrous, with extensive impunctate areas; protibia and tarsus and metatarsus and other characters, variable	**10**
–	Swollen face, with deep sutures; head with punctation distinct, most abundant; elongated maculation in middle of clypeus, narrowing upward, not dilated apically; protibia and tarsus and meta tarsus yellowish; microreticulate area in basal area of the propodeum narrowest, microreticulate sculpture more uniform, regular; puncture line delimiting the supraclypeal plain raised area above, with lateral branches divergent basally	**Ceratina (Ceratinula) turgida (Moure, 1941)** (Brazil: Rio de Janeiro State)
10	Color metallic dark green; large elongated yellow maculation on disc of clypeus; clypeus integument polished, smooth	**Ceratina (Ceratinula) minima Friese, 1908** (Brazil: northern region; Trinidad and Tobago)^2^
–	Color metallic olive-green; yellow band on apical border of clypeus; clypeus integument slight microreticulate	**Ceratina (Ceratinula) piracicabana Schrottky, 1910** (Brazil: São Paulo State)
11	Clypeus with wide light-brown stripe on apical margin (at least 1/6 of clypeus width)	**12**
–	Clypeus with uniform color, without yellow band, sometimes with only the extreme edge lighter	**13**
12	Body metallic green; face uniformly green, without maculation; legs brownish	**Ceratina (Ceratinula) augochloroides Ducke, 1910** (Brazil: Ceará State)
–	Body metallic olive-green; apical margin of clypeus, mandibles, labrum, basal antenna joint, tegula and legs honey-yellow	**Ceratina (Ceratinula) lucidula Smith, 1854** (Brazil States: Pará, Ceará, Minas Gerais, São Paulo; Paraguay)
13	Head with greenish metallic sheen above clypeus; lower area of gena, next to mandibles, pale brownish white; tarsi yellowish brown	**Ceratina (Ceratinula) fulvitarsis Friese, 1925** (Brazil: São Paulo State)
–	Head with bluish metallic sheen above clypeus; lower area of gena near mandibles, of same color as rest of gena; meso- and metarsi dark brown	**Ceratina (Ceratinula) immaculata Friese, 1910** (Brazil: Pará State)

## Conclusions

As stressed by Moure (1941:78–83), the pattern of yellow maculation is extremely important for distinguishing many species of Ceratina (Ceratinula), and most species were previously described were based on differences in the locations of the yellow maculation, especially on the head and legs. Integumental macrosculpture is also considered an important character in a species diagnosis, but primarily for species without yellow maculation. The yellow maculation is more useful for distinguishing females, as the patterns of yellow maculation of males are very similar among species.

With the exception of C. (C.) combinata and C. (C.) minima, which were described from and known only from male specimens, the Brazilian fauna of Ceratina (Ceratinula) has been described based on females, with shape and presence or absence of these yellow maculations explained in the original descriptions.

The identification key presented above is probably not exhaustive, as there are likely to be many species that have not yet been described or recorded in Brazil, but are known from neighboring countries. Therefore, we recommend that the characters be verified in the original descriptions and with a reference collection or type material, in order to more confidently assign species names.

Cockerell (1912) described C. (Ceratinula) manni based on six female specimens collected in Guarabira, Paraíba, Brazil. Until the present contribution, in addition to the type locality, the species had been reported in only two states in northeastern Brazil and from a limited number of specimens: Paraíba, near the coast and in the Atlantic Forest Domain, in Mamanguape (2 females and 2 males) (Aguiar and Martins 2003); three localities in and near João Pessoa (in the unpublished Master’s dissertation of Cruz 2013); and in Cacimba de Dentro, a city in the Caatinga Domain, but near the type locality (1 female) (Zanella and Martins 2005); and the state of Rio Grande do Norte, in Serra Negra do Norte, in the middle of Seridó, one of the driest regions in the Caatinga Domain (2 females and 1 male) (Zanella 2000, 2003). The new geographical records (Bahia: Amélia Rodrigues, Milagres, Maracás; Ceará: Crato; and Piauí: Caracol) expand its distribution considerably to the west and south, but still in the Caatinga Domain or in an ecotone with the Atlantic Forest (open dry diagonal), e.g., Amélia Rodrigues in Bahia (Fig. 10). The new records clearly establish the distribution of C. (Ceratinula) manni in the entire semiarid region of northeastern Brazil (Fig. 10), extending to nearby open-vegetation areas.

Several unidentified specimens of Ceratina (Ceratinula) have been reported in local inventories of bee faunas (some of them cited in the Introduction). Although we have not yet had access to the specimens collected in these surveys, this suggests that the species richness is higher than presently recorded, and that much more collection effort and taxonomic work is necessary to gain a comprehensive knowledge of the diversity and distribution patterns within the subgenus.

Apifaunistic surveys are particularly important sources of floral records for bees on their host plants (Sabino et al. 2011), leveraging data from direct field observations. Surveys also extend taxonomic and biogeographic knowledge, especially regarding species distributions, and are important for assessing pollinator abundance, local richness, and geographic and temporal variations therein, as well as for proposing conservation actions. The only flower records for C. (Ceratinula) manni are for *Stemodia foliosa* Benth. (Scrophulariaceae) from Amélia Rodrigues (Bahia); and for *Indigofera microcarpa* Desv. (Fabaceae) and *Richardia grandiflora* (Cham. et Schl.) Stend. (Rubiaceae) from Serra Negra do Norte (Rio Grande do Norte) and sites in and near João Pessoa in Paraíba (Cruz 2013) (Fig. 10).

## Supplementary Material

XML Treatment for Ceratina (Ceratinula)

XML Treatment for Ceratina (Ceratinula) fioreseana

XML Treatment for Ceratina (Ceratinula) manni

## Footnotes

Ceratina combinata Friese, 1910 was not included in the key because it was described based only on a male specimen, which according to the author himself may be the male of C. muelleri Friese, 1910
